# Report of the Homœopathic Hospital at Melbourne, 1888

**Published:** 1889-01

**Authors:** 


### Report of the Homœopathic Hospital at Melbourne, 1888.

Our thanks are due to Dr. Bouton for a copy of this report, showing an active, useful hospital; its building is soon to be enlarged, means for this purpose having been donated by a generous friend.

